# Validation and reliability of the Thai version of the glasgow antipsychotic side-effect scale (GASS) in patients with schizophrenia

**DOI:** 10.3389/fphar.2026.1899860

**Published:** 2026-09-01

**Authors:** Nattida Wunha, Siriprapa Maksuk, Sutrak Pilakanta, Sirivipa Jekpoo, Porntip Apiwatnakorn, Mark Taylor, Tuanthon Boonlue, Tinakon Wongpakaran, Rewadee Jenraumjit

**Affiliations:** 1 Department of Pharmaceutical Care, Faculty of Pharmacy, Chiang Mai University, Chiang Mai, Thailand; 2 Nursing Department, Maharaj Nakorn Chiang Mai Hospital, Chiang Mai University, Chiang Mai, Thailand; 3 Department of Psychiatry, Faculty of Medicine, Chiang Mai University, Chiang Mai, Thailand; 4 Department of Pharmacy, Suan Prung Psychiatric Hospital, Chiang Mai, Thailand; 5 Griffith University, Brisbane, QLD, Australia; 6 The Edinburgh Practice, Edinburgh, United Kingdom; 7 Faculty of Pharmaceutical Sciences, Ubon Ratchathani University, Ubon Ratchathani, Thailand

**Keywords:** antipsychotics, glasgow antipsychotic side-effect scale (GASS), reliability, schizophrenia, side effects, validity

## Abstract

**Objective:**

To examine the validity and reliability of the Thai version of Glasgow Antipsychotic Side-effect Scale (GASS) in patients with schizophrenia.

**Method:**

A cross-sectional analytical study was conducted among patients aged 20 years and older who had been receiving continuous antipsychotic treatment for at least 3 months and attended Suan Prung Psychiatric Hospital and Maharaj Nakorn Chiang Mai Hospital, Chiang Mai, Thailand. The GASS was translated into Thai using a forward–backward translation process. Psychometric properties were evaluated, including construct validity assessed by confirmatory factor analysis (CFA, n = 72), concurrent validity examined through correlations between GASS scores and the Thai Perceived Stress Scale-10 (T-PSS-10), EQ-5D-5L, and the Pharmacist Medication Adherence Scale (PMAS), as well as reliability assessed by internal consistency and test–retest reliability (n = 53, with a 7-day interval).

**Results:**

The CFA model demonstrated marginal fit to the empirical data (CFI = 0.919, TLI = 0.909, RMSEA = 0.059, SRMR = 0.141). GASS scores were positively correlated with T-PSS-10 (Spearman’s rho = 0.41) and negatively correlated with EQ-5D-5L utility scores, EQ-5D-5L visual analogue scale scores, and PMAS (Spearman’s rho = −0.64, −0.39, and −0.24, respectively), with statistical significance (p < 0.05). The Thai version of the GASS demonstrated good internal consistency (Cronbach’s alpha = 0.83) and good test–retest reliability (ICC = 0.797, p < 0.05).

**Conclusion:**

The Thai version of the GASS shows preliminary evidence of acceptable validity and reliability and can be appropriately used to assess antipsychotic side effects in patients with schizophrenia in both clinical practice and research settings.

## 1 Introduction

Schizophrenia is characterized by abnormalities in brain function that manifest as disturbances in thought, emotion, and behavior with varying levels of severity. It represents a significant public health concern worldwide. The disorder typically follows a chronic course, often requiring long-term management, although periods of symptomatic improvement may occur (Department of Mental Health and Ministry of Public Health, 2017; Taylor and Jauhar, 2019). According to the World Health Organization 2024, schizophrenia affects approximately 24 million people worldwide, which corresponds to about one in 300 individuals globally (World Health Organization, 2025).

The treatment of schizophrenia usually involves the use of antipsychotic medications, which are often classified into two groups: first-generation antipsychotics (FGAs) and second-generation antipsychotics (SGAs). FGAs are commonly associated with adverse effects such as Extrapyramidal Symptoms (EPS). In contrast, SGAs demonstrate broader therapeutic efficacy and are associated with a lower incidence of EPS. However, these agents may lead to other important side effects, including weight gain, hyperglycemia, dyslipidemia, and metabolic syndrome, which can increase the risk of developing cardiovascular disease (Chanthawong et al., 2017).

Side effects associated with antipsychotic medications predict medication adherence among patients with schizophrenia (Chaiphet, 2024), and those who experience side effects tend to have lower levels of medication adherence. Nonadherence to antipsychotic therapy among patients with schizophrenia may consequently lead to relapse of the illness (Taylor and Jauhar, 2019; Chaiphet, 2024).

Side effects associated with antipsychotic medications can be overlooked because the symptoms may be subtle or not immediately recognized as being related to medication use. A ‘rule’ in medicine is that you don’t see what you don’t look for. Therefore, systematic monitoring and assessment of side effects related to antipsychotic medications are essential for effective care for patients with schizophrenia.

The assessment of side effects associated with psychotropic medications can be categorized into multidimensional evaluation tools, which are suitable for clinical use as they cover commonly reported symptoms and allow patients to perform self-assessment. Currently, several assessment tools have been developed and are widely used, including the Barnes Akathisia scale, Liverpool University Neuroleptic Side Effect Rating Scale (LUNSERS), and the Glasgow Antipsychotic Side-effect Scale (GASS) (Endriyani et al., 2019; van Strien et al., 2015). These assessment tools have an acceptable level of reliability, with reported Cronbach’s alpha values greater than 0.70.

The 22-item GASS, created by Waddell and Taylor, usually takes five to 10 minutes to complete (van Strien et al., 2015). It has been translated and shown to be valid and reliable in a number of studies (Waddell and Taylor, 2008). The GASS was theoretically created as a unidimensional construct to represent a patient’s overall, subjective side-effect burden. Since clinician-rated scales frequently underestimate subclinical suffering that directly affects medication adherence, it is crucial to capture the patient’s actual experience of drug tolerability in accordance with WHO Patient-Reported Outcome (PRO) frameworks. The 22-item GASS provides a brief, comprehensive, self-administered alternative for instruments like the Barnes Akathisia Scale, which only focuses on one symptom domain, and the LUNSERS, which is extensive (41 items), and limits utility in busy Thai outpatient clinics.” GASS has not yet been translated into Thai, and its validity and reliability have not been evaluated in Thailand. Therefore, this study aimed to translate GASS into Thai and examine its psychometric properties to determine its suitability for use in Thailand.

## 2 Methods

### 2.1 Study design and participants

This was a quantitative study using a cross-sectional analytical design. The study population consisted of outpatients with schizophrenia who received treatment at Suan Prung Psychiatric Hospital and Maharaj Nakorn Chiang Mai Hospital between October 2025 and January 2026.

The inclusion criteria were patients aged 20 years or older; diagnosed with schizophrenia according to the diagnostic criteria of the International Classification of Diseases, 10th Revision (ICD-10); receiving at least one antipsychotic medication; attending scheduled follow-up visits and continuously receiving medication for at least 3 months (concomitant medications were not considered and continuous use of the same medication was not required); and having sufficient understanding to complete the questionnaire, including the ability to speak, read, and write Thai.

The exclusion criteria were receiving electroconvulsive therapy; having neurological conditions including epilepsy, movement disorders, intellectual disability, or dementia; presenting with active symptoms of delusions, hallucinations, or mania; and using psychoactive substances such as alcohol, amphetamines, or cannabis, regardless of the diagnosis according to ICD-10.

This research project was approved by the Human Research Ethics Committee of Suan Prung Hospital (Research project number: SPH. IRB026/2568 SCs_exp; approval date: 22 October 2025) and Maharaj Nakorn Chiang Mai Hospital (Research project number: 0513; approval date: 3 October 2025).

### 2.2 Sample size calculation

In this study, 105 patients with schizophrenia receiving antipsychotic medications were recruited, including 70 patients from Suan Prung Psychiatric Hospital and 35 patients from Maharaj Nakorn Chiang Mai Hospital. The sample size was calculated according to (Costello and Osborne, 2005; Hair et al., 2013). Using the rule-of-thumb criterion with a ratio of 5:1 (number of participants per questionnaire item), which represents the minimum requirement. Since the GASS consists of 21 items, the required sample size was therefore determined to be at least 105 participants (Costello and Osborne, 2005; Hair et al., 2013).

### 2.3 Instruments used in the study

This study employed three types of research instruments: a general information form, the GASS for assessing adverse effects from antipsychotic medications, and an instrument used to examine concurrent validity.

1. General information was obtained from the electronic medical records of Suan Prung Psychiatric Hospital and Maharaj Nakorn Chiang Mai Hospital**.**
2. GASS (Waddell and Taylor, 2008; Bokhari et al., 2023; Rodolico et al., 2022) The scale consists of 22 items. Twenty items assess symptoms experienced during the past 1 week, while the remaining two items (changes in menstrual cycle and weight gain) assess symptoms occurring during the past 3 months. Respondents rate the frequency of each symptom on a scale ranging from 0 to three points. The total score is categorized into three levels of severity: none/mild (0–21 points), moderate (22–42 points), and severe (43–63 points). In addition, the level of distress associated with each symptom is assessed on a scale ranging from 1 to 10 points.
3. Three validity instruments were used in this study, including
  3.1 The Thai version of the Perceived Stress Scale (T-PSS-10) (Wongpakaran and Wongpakaran, 2010) This study demonstrated a Cronbach’s α = 0.7224.
  3.2 The Thai version of the EQ-5D-5L quality of life questionnaire (EQ-5D-5L. 2025, 2025; Pattanaphesaj and Thavorncharoensap, 2017). This study demonstrated a Cronbach’s α = 0.7855.
  3.3 The Patient Medication Adherence Scale (PMAS). This study demonstrated a Cronbach’s α = 0.6501.

### 2.4 Translation and validation of the GASS

Permission to translate the GASS into Thai was obtained from Mark Taylor, the original developer of the instrument. After permission was granted, the forward translation was performed by a Thai clinical expert with proficiency in English and a native English-speaking translator. The two translated versions were then synthesized into a single Thai version. Subsequently, back-translation was conducted by a British clinical expert and a native Thai-speaking translator. The back-translated version was then sent to the original developer of GASS to verify its consistency with the original English version. To establish semantic, conceptual, and experiential equivalence in the Thai healthcare context, the synthesized version was reviewed by a multidisciplinary expert panel comprising one psychiatric pharmacist, one psychiatric nurse, and two psychiatrists, all with clinical and academic expertise in psychopharmacology and schizophrenia care. The panel refined wording and clinical language to ensure clarity and comprehensibility for ordinary patients.

The panel also evaluated content validity using the Content Validity Index (CVI). Item-level CVI (I-CVI) values ranged from 0.83 to 1.00, and the scale-level CVI (S-CVI/Ave) was 0.92, indicating excellent agreement among experts regarding item relevance and clarity.

Finally, during the initial data collection phase, the psychiatric pharmacist and psychiatric nurse cross-verified that patients could easily understand the self-reported items without semantic ambiguity, further supporting the equivalence of the Thai version for use in clinical and research settings. Permission to use the EQ-5D-5L was obtained through the website of the EuroQol Group.

Data collection was conducted at the psychiatric outpatient clinics of Suan Prung Psychciatric Hospital and the psychiatric clinic at Maharaj Nakorn Chiang Mai Hospital. All three assessment instruments were administered on the same day. However, the PMAS was evaluated by a pharmacist. In addition, participants were contacted by telephone to complete the GASS again 1 week after the initial assessment to evaluate test-retest reliability. In addition, participants were contacted by telephone to complete the GASS again 1 week after the initial assessment to evaluate test-retest reliability. Crucially, to preserve the integrity of the stability assumption, participants were formally screened during the second assessment to confirm that their psychiatric clinical condition had remained stable, and that their antipsychotic medications, dosages, and administration regimens were completely unchanged during the intervening week.

### 2.5 Outcomes and statistical analysis

The primary outcome of this study was the validity and reliability of the Thai-translated version of GASS in Thai patients with schizophrenia.

Data was analyzed using Stata version 14.0 and Mplus 9.1. Descriptive data were reported as frequencies and percentages for categorical variables, while continuous variables were presented as means and standard deviations (SD). Scores from the assessment scale were reported as mean, SD, median, interquartile range (IQR), and minimum–maximum values. Comparisons of GASS scores between hospital groups, types of antipsychotic medications, and dosage levels were performed using the Wilcoxon rank-sum test. Assumptions of normality were formally tested using skewness and kurtosis indices, which indicated significant departures from normality. Multicollinearity was assessed through variance inflation factors (VIF), with all values <2, suggesting no problematic collinearity. Missing data were minimal (<5%) and handled using pairwise deletion, consistent with COSMIN recommendations. For criterion validity, Spearman’s rank correlation was used due to the non-normal distribution of ordinal data. For structural validity, Confirmatory Factor Analysis (CFA) was conducted to test the hypothesized unidimensional construct of the GASS. CFA employed the Weighted Least Squares Mean and Variance adjusted (WLSMV) estimator, justified by the ordered categorical nature of the GASS response scale (0–3 Likert items) and the observed non-normality. Model fit was determined using the following fit indices: Comparative Fit Index (CFI) ≥ 0.95, Tucker–Lewis Index (TLI) ≥ 0.90, Root Mean Square Error of Approximation (RMSEA) ≤ 0.06, and Standardized Root Mean Square Residual (SRMR) ≤ 0.08 (Xia and Yang, 2019). Criterion validity was evaluated by examining the correlations between scores of the GASS and those of the T-PSS-10, EQ-5D-5L, and PMAS using Spearman’s rank correlation. The reliability of the scale was assessed by evaluating internal consistency using Cronbach’s alpha, with a criterion value of ≥0.70 (Bland and Altman, 1997). Test–retest reliability was also evaluated using the Intraclass Correlation Coefficient (ICC) based on a two-way mixed-effects model for GASS. Poor reliability is indicated by ICC values less than 0.50, moderate reliability by values between 0.50 and 0.75, good reliability by values between 0.75 and 0.90, and excellent reliability by values greater than 0.90 (Koo and Li, 2016).

## 3 Results

### 3.1 Baseline characteristics

**FIGURE 1:**
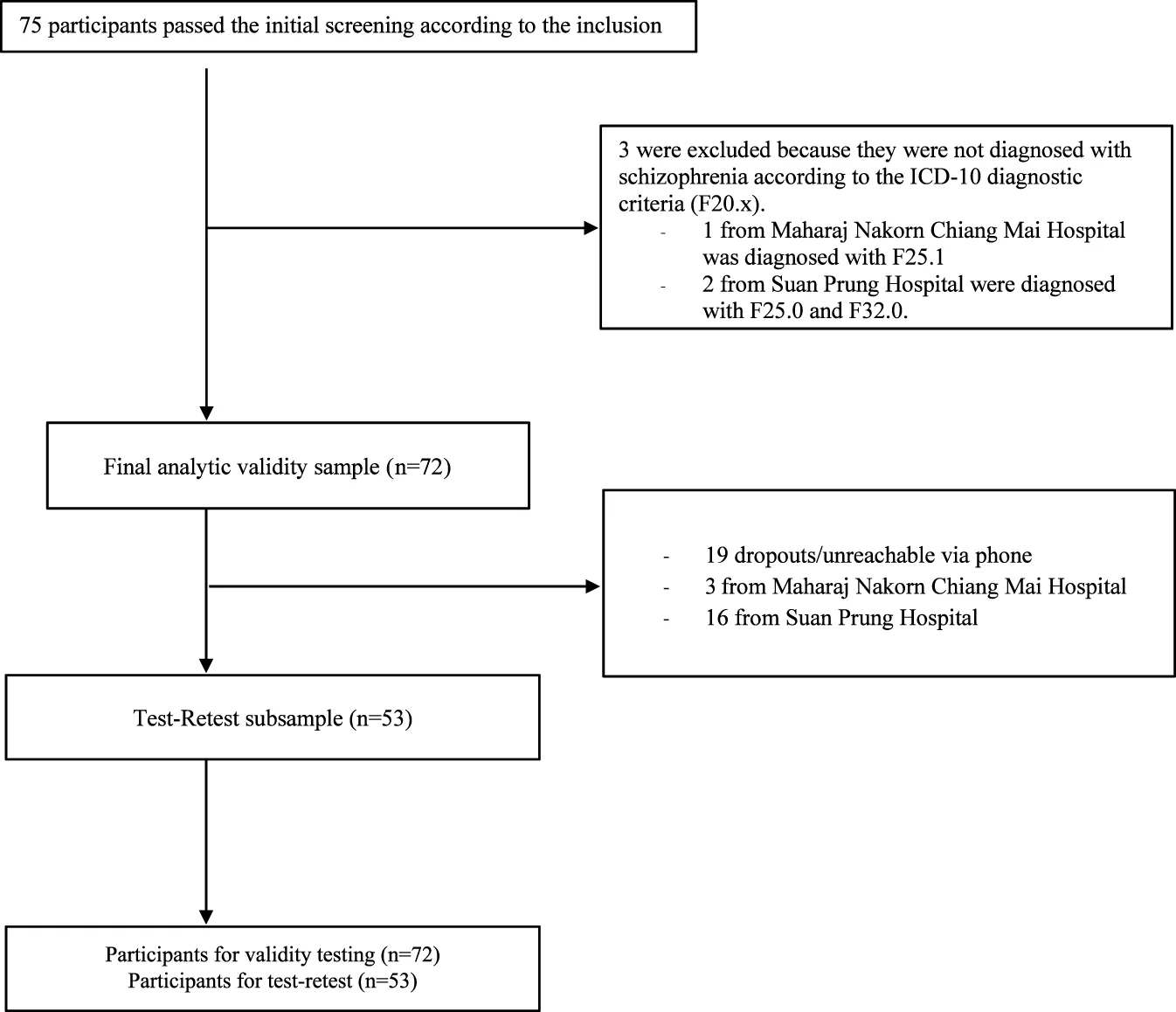
Flow diagram of recruitment process. Participants for validity testing (n=72). Participants for test-retest (n=53).

The recruitment of study participants is illustrated in Figure 1, and the baseline characteristics of the participants are presented in Table 1.

**TABLE 1:** Baseline characteristics of the participants.

General characteristics		Total sample (n = 72) n (%)	​
Age	<50 years >50 years	39 (54.2) 33 (45.8)	​
Body Mass index (BMI)	≤18.4 kg/m^2^ 18.5–22.9 kg/m^2^ 23.0–24.9 kg/m^2^ ≥25.0 kg/m^2^	1 (1.4) 16 (22.2) 13 (18.1) 42 (58.3)	​
Sex	Male Female	30 (41.7) 42 (58.3)	​
Marital status	Single Married Divorced	55 (76.4) 12 (16.7) 5 (6.9)	​
Education level	Primary education Lower secondary education Upper secondary education Bachelor’s degree Other	8 (11.1) 12 (16.7) 18 (25.0) 23 (31.9) 11 (15.3)	​
Health insurance scheme	Universal coverage scheme Social security scheme Civil servant medical benefit scheme Other	43 (59.7) 9 (12.5) 7 (9.7) 13 (18.1)	​
Alcohol consumption	Never Current Former	63 (87.5) 6 (8.3) 3 (4.2)	​
Smoking status	Never Current Former	50 (69.4) 21 (29.2) 1 (1.4)	​
Psychiatric comorbidities	None Depression Anxiety disorder Bipolar disorder Other	71 (98.6) N/A N/A N/A 1 (1.4)	​
Comorbidities	None Diabetes mellitus Cardiovascular and cerebrovascular diseases Dyslipidemia Obesity Other	44 (61.1) 5 (6.9) N/A 13 (18.1) N/A 15 (20.8)	​
Type of schizophrenia (ICD-10)	F20.0 paranoid schizophrenia F20.1 hebephrenic schizophrenia F20.3 undifferentiated schizophrenia F20.4 post schizophrenic depression F20.5 residual schizophrenia F20.6 simple schizophrenia F20.8 other schizophrenia F20.9 schizophrenia, unspecified	50 (69.4) 2 (2.8) 1 (1.4) 3 (4.2) 2 (2.8) 1 (1.4) 3 (4.2) 10 (13.9)	​
Antipsychotics treatment regimen	Monotherapy Polypharmacy	31 (43.1) 41 (56.9)	​
Type of antipsychotic medication	FGAs SGAs FGAs and SGAs	15 (20.8) 34 (47.2) 23 (31.9)	​
Route of administration	Oral Injection Oral and injection	53 (73.6) 4 (5.6) 15 (20.8)	​
History of misses medication	No Yes	46 (63.9) 26 (36.1)	​
Medication adherence	Poor adherence Good adherence	2 (2.8) 70 (97.2)	​

The participants had a mean age of 47.97 ± 11.77 years, with 54.2% of participants being under 50 years old. Most of the participants were female, accounting for 58.3% (n = 42). In terms of the type of schizophrenia, The vast majority were diagnosed with paranoid schizophrenia (F20.0), representing 69.4% of the participants, Regarding the antipsychotic treatment regimen, nearly half of the patients (47.2%) were treated with SGAs alone, 31.9% received only first-generation antipsychotics (FGAs), and 31.94% were prescribed a combination of both FGAs and SGAs. Additionally, a polypharmacy regimen was more common 56.9% than monotherapy (43.1%).

The comparison of side-effect scores assessed using the GASS between Maharaj Nakorn Chiang Mai Hospital and Suan Prung Psychiatric Hospital is shown in Table 2. The mean scores were 11.94 ± 10.95 and 12.18 ± 8.24, respectively. These scores fall within the range of 0–12 points, indicating no or mild side effects. No statistically significant difference was observed between the two hospitals (p = 0.639).

**TABLE 2:** Comparison of GASS scores of Suan Prung Psychiatric Hospital and Maharaj Nakorn Chiang Mai Hospital.

Study setting	Mean	SD	p-value
Maharaj nakorn chiang mai hospital (n = 16)	11.94	10.95	0.639
Suan prung hospital (n = 56)	12.18	8.24	

In addition, the associations between types of antipsychotic medications, antipsychotic formulations, and routes of administration with GASS scores were analyzed. No statistically significant differences were found among the groups (p > 0.05), as presented in Table 3.

**TABLE 3:** Association between type of antipsychotic medication, formulation, and route of administration and GASS scores.

​	Mean	SD	Median (IQR)	p-value
Type of antipsychotic medication				
FGAs* (n = 15)	12.47	8.855	13 (18)	0.240
SGAs* (n = 34)	10.41	8.180	8.5 (10)	
FGAs and SGAs (n = 23)	14.43	9.534	12 (14)	
Antipsychotic formulation				
Monotherapy* (n = 31)	10.45	9.011	8 (10)	0.096
Polypharmacy* (n = 41)	13.39	8.576	13 (10)	
Route of administration				
Oral (n = 53)	10.66	8.340	9 (10)	0.056
Injection** **(n = 4)	15.00	4.080	13.5 (5)	
Oral and injection** **(n = 15)	16.53	10.09	15 (15)	

* FGAs, First generation antipsychotic; SGAs, Second generation antipsychotic, Monotherapy = use of a single antipsychotic medication, Polypharmacy = use of multiple antipsychotic medications.

### 3.2 Characteristics of study instruments

The distribution of total scores from the GASS, T-PSS-10, EQ-5D-5L, and PMAS questionnaires is presented in Table 4. Floor and ceiling effects were evaluated for the total GASS score to determine the scale’s content validity and discriminative capabilities. A threshold of >15% was utilized to define the presence of significant floor or ceiling effects. No ceiling effect was detected, as no participant (0%) achieved the maximum theoretical score of 63, with the highest recorded score being 42. Conversely, a minor floor effect was observed, with eight out of 72 participants (11.1%) scoring the absolute minimum score of 0.

**TABLE 4:** Distribution of total scores from the GASS, T-PSS-10, EQ-5D-5L, and PMAS (n = 72).

Statistic	GASS	TPSS-10	EQ-5d-5L			PMAS	
	Total	Total	Utility	VAS	Total	Self-rated	Pharmacist-rated
Mean	12.13	13.07	0.8672	75.76	28.39	9.340	8.972
SD	8.825	6.363	0.1715	18.32	2.730	1.321	1.547
Min, max	0,42	0,25	0.0079,1	30,100	18,30	3,10	1,10
Skewness*	1.056	−0.3604	−2.390	−0.5553	−2.321	−2.788	−2.504
Kurtosis**	4.656	2.407	10.75	2.347	8.192	11.43	11.76

*Skewness = measure of the asymmetry of a distribution, ** Kurtosis = measure of the peakedness of a distribution compared with a normal distribution.

### 3.3 Validity

#### 3.3.1 Construct validity

Construct validity was assessed using CFA with the WLSMV estimator. A standardized factor loading threshold of >0.50 was established to indicate an acceptable relationship between an item and the underlying latent construct. The results indicated that the model demonstrated a marginal fit with the empirical data (CFI = 0.919, TLI = 0.909, RMSEA = 0.059, and SRMR = 0.141). “The psychometric evaluation also demonstrated strong reliability and acceptable convergent validity for the Thai version of the GASS. The composite reliability (CR) was excellent (0.93), indicating that the items consistently measure the underlying construct of subjective side-effect burden. The average variance extracted (AVE) was 0.47, slightly below the conventional threshold of 0.50, suggesting moderate convergent validity. This finding reflects some residual measurement error across items, which is common in patient-reported outcome measures with heterogeneous symptom content. Overall, the results support the internal consistency and structural validity of the instrument, while highlighting the need for further investigation of item refinement in larger samples.

#### 3.3.2 Concurrent validity

GASS scores showed a moderate positive correlation with T-PSS-10 (ρ = 0.4086, P<0.001) (Table 5), indicating that individuals with greater antipsychotic side effects tended to report higher perceived stress. In contrast, GASS scores were significantly negatively correlated with quality of life measured by EQ-5D-5L, both in terms of the utility score (ρ = −0.6435, p < 0.001) and the VAS score (ρ = −0.3933, P<0.001), suggesting that increased adverse effects were associated with poorer quality of life (Table 5). For PMAS, a weak negative correlation was observed between GASS scores and the total score (ρ = −0.2376, P=0.044) (Table 5). (Selvanathan et al., 2020).

**TABLE 5:** Correlations between total GASS score and t-pss-10, EQ-5D-5L, and PMAS scores (n = 72).

TPSS-10	​	EQ-5d-5L		​	PMAS	
	​	Utility	VAS	​	Self-rated	Pharmacist-rated
GASS	0.4086	−0.6435	−0.3933	−0.2376	−0.1321	−0.1593
p-value	<0.001	<0.001	<0.001	0.044	0.269	0.181

### 3.4 Reliability

#### 3.4.1 Internal consistency

The Cronbach’s alpha for the entire scale was 0.8347, indicating good internal consistency reliability (Cronbach’s alpha >0.70).

#### 3.4.2 Test–retest reliability

Test–retest reliability was assessed using the Intraclass Correlation Coefficient (ICC) (two-way mixed-effects model, absolute agreement). The total GASS score demonstrated a good level of reliability with ICC = 0.797 (0.75–0.90) (Table 6). Most item-level scores showed moderate to excellent reliability, with several items having ICC values ≥0.75 (Koo and Li, 2016). Specifically, item 9 (ICC = 0.234), item 10 (ICC = 0.298), item 14 (ICC = 0.105), item 17 (ICC = 0), item 18 (ICC = 0) demonstrated low factor loadings below the established threshold, indicating weak structural associations with the broad unidimensional side-effect factor.

**TABLE 6:** Test–retest reliability analysis of item scores of the GASS (n = 53).

Items	ICC	p-value	95% CI-interval
1. I felt sleepy during the day	0.675	<0.001	0.497–0.799
2. I felt drugged or like a zombie	0.570	<0.001	0.356–0.727
3. I felt dizzy when I stood up and/or have fainted	0.377	0.003	0.118–0.587
4. I have felt my heart beating irregularly or unusually fast	0.680	<0.001	0.504–0.801
5. My muscles have been tense or jerky	0.778	<0.001	0.644–0.866
6. My hands or arms have been shaky	0.769	<0.001	0.630–0.860
7. My legs have felt restless and/or I couldn’t sit still	0.636	<0.001	0.446–0.772
8. I have been drooling	0.741	<0.001	0.589–0.842
9. My movements or walking have been slower than usual	0.234	0.045	(-0.0378)-0.474
10. I have had uncontrollable movements of my face or body	0.298	0.014	0.0330–0.524
11. My vision has been blurry	0.768	<0.001	0.631–0.859
12. My mouth has been dry	0.734	<0.001	0.581–0.837
13. I have had difficulty passing urine	0.544	<0.001	0.324–0.709
14. I have felt like I am going to be sick or have vomited	0.105	0.229	(-0.173)-0.365
15. I have wet the bed	0.386	0.002	0.138–0.590
16. I have been very thirsty and/or passing urine frequently	0.877	<0.001	0.796–0.927
17. The areas around my nipples have been sore and swollen	0.000	0.500	(-0.268)-0.268
18. I have noticed fluid coming from my nipples	0.000	0.500	(-0.268)-0.268
19. I have had problems enjoying sex	0.596	<0.001	0.391–0.745
20. Men only: I have had problems getting an erection	0.757	<0.001	0.515–0.889
21. Women only: I have noticed a change in my periods	0.914	<0.001	0.829–0.958
22. Men and women: I have been gaining weight	0.925	<0.001	0.874–0.956
Total	0.797	<0.001	0.672–0.877

## 4 Discussion

No significant differences in GASS scores were observed between the two hospitals, supporting the combined analysis of the data. GASS scores were concentrated at lower levels, whereas EQ-5D-5L and PMAS scores were concentrated at higher levels. This distribution reflects a population of patients with stable conditions and supports the use of non-parametric statistical methods.

No significant differences in GASS scores were observed between FGAs and SGAs, monotherapy and polypharmacy, or oral and long-acting injectable formulations. These findings may reflect individualized treatment approaches in routine clinical practice, in which antipsychotic regimens are continuously adjusted to optimize efficacy while minimizing side effects. Although different antipsychotic classes and formulations are associated with distinct adverse-effect profiles, dose optimization, ongoing clinical monitoring, and concomitant treatments may reduce differences in the overall burden of patient-reported side effects as measured by the GASS. Similarly, patients receiving polypharmacy may receive lower doses of individual agents to improve tolerability, whereas those receiving monotherapy may require higher doses to maintain symptom control, resulting in comparable GASS scores between groups. In addition, long-acting injectable and oral antipsychotics share similar pharmacological properties, which may contribute to similar levels of self-reported side effects despite differences in administration routes. Antipsychotic-related side effects are also likely influenced by multiple factors beyond medication class or formulation alone, including illness severity, treatment duration, concomitant medications, and individual variability in drug response.

Although the GASS encompasses a wide range of symptom domains, factor analysis indicated that the items predominantly reflect a single underlying construct, supporting its essentially unidimensional nature. The CFA using the WLSMV estimator demonstrated marginal model fit, with the CFI slightly below the recommended threshold and the SRMR elevated (0.141). Reliability indices were strong, with composite reliability (CR = 0.93) indicating excellent internal consistency, while the average variance extracted (AVE = 0.47) suggested only moderate convergent validity. Taken together, this pattern reflects residual item-level misfit: the scale is reliable overall, but heterogeneity among side-effect items may limit convergent validity. Elevated SRMR values typically signal residual correlations not fully explained by a strict unidimensional model, which in the case of the GASS likely arise from the diverse nature of antipsychotic side effects, ranging from metabolic changes such as weight gain to neurological symptoms like sedation or abnormal movements (Rodolico et al., 2022). Given that the GASS is a symptom-based patient-reported outcome measure, such deviations are not unexpected, as individual items may share variance beyond the general construct of subjective burden. We therefore interpret the elevated SRMR as reflecting item heterogeneity rather than a fundamental flaw in construct validity. Importantly, other fit indices (RMSEA, TLI, CFI) remained within acceptable ranges, supporting the structural validity of the Thai GASS for clinical monitoring purposes.

Concurrent validity demonstrated a moderate positive correlation with perceived stress and a negative correlation with quality of life, which is consistent with previous literature (Kretchy et al., 2015). Thai GASS exhibited a weak negative correlation with the PMAS (Spearman’s rho = −0.24). This weak association underscores that medication adherence in schizophrenia is a highly complex, multi-determined behavioral outcome, rather than a simple linear reaction to side-effect severity (Guo et al., 2023). In the Thai cultural context, adherence is heavily buffered by strong community networks and routine interventions, which may override the urge to discontinue medication despite physical discomfort. Furthermore, this weak association highlights the inherent tension between the psychometric assumption of unidimensionality and the clinical heterogeneity of antipsychotic side effects. Mathematically, the CFA attempts to collapse all side effects into a single latent burden score. However, clinically, a patient experiencing severe akathisia might discontinue medication immediately, whereas a patient experiencing gradual weight gain might remain fully adherent despite a high cumulative GASS score. Therefore, a unidimensional total score may dilute specific adherence-altering clinical signals. In real-world schizophrenia management, the GASS should not solely be used to generate a total score, but rather to identify specific heterogeneous domains (e.g., neurological vs. metabolic) that uniquely threaten the therapeutic alliance and long-term treatment adherence.”. Additionally, the Patient Medication Adherence Scale (PMAS) showed a Cronbach’s alpha <0.70. Therefore, other instruments may be needed for assessing medication adherence, such as the Medication Adherence Scale in Thais (MAST). (Aukkarakornkul and Lerkiatbundit, 2023).

GASS demonstrated good internal consistency, with a Cronbach’s alpha of 0.84, meeting the recommended criterion and comparable to findings from studies conducted in other countries such as the Italian (α = 0.81), Greek (α = 0.793), and Japanese Clozapine-specific (α = 0.82) validation cohorts (Rodolico et al., 2022; Maria et al., 2014; Kitagawa et al., 2020). The cross-cultural stability of the GASS suggests that despite diverse ethnic pharmacogenomic profiles and varying healthcare systems, the subjective experience and reporting of antipsychotic side-effect burdens remain remarkably consistent among patients with schizophrenia. The test–retest reliability of the total score was also good (ICC = 0.80), consistent with previous literature (Maria et al., 2014). Although some items showed near-zero or poor reliability (e.g., slower/uncontrollable movements, nipple swelling, fluid discharge, and vomiting)., these symptoms may occur infrequently or vary over time in stable patients, which could reduce the consistency of repeated measurements. Rather than psychometric weakness alone, these reflect clinically highly episodic side effects that naturally fluctuate over a short time, meaning they should be retained for safety monitoring despite low statistical ICCs.

In terms of clinical implications, the use of GASS in outpatient settings should follow an assessment interval consistent with the instrument’s 7-day recall period. The assessment should be conducted at least 1 week after initiating or adjusting antipsychotic medication, and subsequently repeated at 4 weeks, 12 weeks, and 6 months, as well as whenever dosage adjustments occur, to ensure appropriate and continuous monitoring of adverse effects (CG178 N, 2014; Singh et al., 2024). Interpretation of the results should consider both the severity score and the level of distress experienced by patients. A total score of 22 or higher should prompt further clinical evaluation and appropriate management. However, even when the total score is low, additional assessment may be warranted if the patient reports high levels of distress. Although GASS is a self-administered questionnaire, interpretation of the results should be conducted in collaboration with healthcare professionals to guide individualized patient care. Given its brevity and ease of use, the questionnaire may also be completed collaboratively with a psychiatrist or pharmacist to facilitate more accurate assessment and clinical discussion regarding antipsychotic-related side effects.

Although this study provides valuable preliminary psychometric evidence for the Thai version of the GASS, several limitations must be explicitly acknowledged. First, the sample size utilized for the CFA was relatively modest (n = 72). While statistically justified by specific psychometric guidelines for stable populations, a larger sample size would be required to ensure absolute parameter stability and robustness in structural equation modeling. Second, this initial validation focused primarily on construct and concurrent validity, lacking a formal evaluation of convergent and discriminant validity matrices. Consequently, the distinct boundaries of the GASS item clusters require further investigation. Third, due to the limited sample size, we did not assess measurement invariance across key demographic and clinical subgroups, such as gender or types of antipsychotic regimens; therefore, it remains unverified whether the scale measures side-effect constructs identically across these segments. Cross-cultural measurement invariance was not formally tested, and we acknowledge this as a limitation. Future studies should consider evaluating measurement invariance to further strengthen evidence for cross-cultural validity. Fourth, because the GASS is a patient-reported outcome measure, the data are inherently susceptible to recall bias, particularly for items requiring a 3-month retrospective assessment (e.g., changes in weight or menstrual cycles). Finally, the generalizability of our findings is constrained, as the study population was exclusively recruited from two tertiary outpatient settings in northern Thailand and consisted primarily of clinically stable patients. Therefore, these preliminary psychometric properties may not fully reflect the side-effect experiences of acute, inpatient, or geographically diverse schizophrenic cohorts in Thailand. Future multicenter studies with larger, stratified samples are recommended to address these gaps.

While this study successfully provides preliminary structural validation using Classical Test Theory (CTT) and CFA, future research should interpret GASS within modern psychometric frameworks, such as Item Response Theory (IRT) or Rasch latent trait modeling. Applying IRT would allow researchers to precisely map the item difficulty and discrimination parameters of GASS. For example, it could determine which specific side effects (e.g., lactation vs. slower movements) provide the highest psychometric information across different severity spectrums of the latent side-effect trait. Transitioning to IRT frameworks will ultimately help refine the instrument, potentially establishing differential weighting for items that are clinically critical but psychometrically distinct.

## 5 Conclusion

The Thai version of the GASS demonstrates preliminary evidence of validity and reliability; however, further validation with larger samples and advanced psychometric approaches is recommended. Clinicians and patients also reported that the Thai GASS was an easy instrument to use and train on, allowing both cross-sectional measures of adverse side effects and their severity, but also longitudinal follow-up of identified problems. Additionally, use of GASS supports structured professional judgement for the clinician, acting as an ‘aide-memoire’, and facilitates enquiry of tricky topics such as sexual dysfunction. Overall, these findings support that the Thai GASS has appropriate validity and reliability for assessing side effects of antipsychotic medications in both research and clinical practice.

## Data Availability

The raw data supporting the conclusions of this article will be made available by the authors, without undue reservation.
